# “Oh, the places you'll go”: The psychological consequences of omission and misrepresentation for Native children

**DOI:** 10.1111/cdev.14193

**Published:** 2024-10-30

**Authors:** Stephanie A. Fryberg, Arianne E. Eason

**Affiliations:** ^1^ Northwestern University Evanston Illinois USA; ^2^ University of California, Berkeley Berkeley California USA

## Abstract

All children navigate the world by searching for information in their sociocultural contexts (e.g., schools, media, laws) to make sense of their experiences and potential futures. In doing so, Native children, however, must contend with the legacy and ongoing oppression of their Peoples, communities, and ways of being. In this manuscript, we highlight how sociocultural contexts stemming from settler colonialism undermine Native futures. We detail how settler colonialism, specifically its manifestation in acts of omission and misrepresentation, impacts children's self and identity development, and intergroup relations. We close by acknowledging the importance of understanding development in context, and the power that the representational landscape can have for promoting paths toward positive child development and greater equity.

In the process of developing a concept of self, all children navigate the world by searching, both consciously and unconsciously, for self‐relevant information (Fryberg & Townsend, 2008; Wagner, 1997). They look out into the world for people who look like them and who have similar characteristics and experiences (Oyserman & Fryberg, 2006 ). This information helps children to develop answers to questions such as, “Who am I,” “How can I make sense of my experiences,” and “What might my future look like.” This process of self‐development and understanding is not necessarily one of individual contemplation, but instead occurs as a result of engaging in social interactions with others and contending with ideas about who and what is good, right and moral (Markus & Wurf, 1987; Moscovici, 1984; Moscovici, 1988; Wagner, 1997). In other words, for all children, sociocultural contexts (e.g., schools, media, laws, and norms) influence their thoughts, feelings, and behaviors. For Native children (i.e., children who identify as Native), the broader US sociocultural context creates additional challenges to navigate. As it currently stands and as is highlighted throughout this special issue, developmental science has a long way to go toward understanding, characterizing, and shedding light on the unique experiences of Native children.

In particular, the challenges that Native children face are rooted in an ongoing process of settler colonialism. Specifically, the United States is a settler colony whose very existence required usurping the lands and oppressing the social and cultural structures of the original inhabitants, the indigenous peoples—Native People—who have resided here since time immemorial. In order to carry out and maintain this process, settlers and their descendants have long constructed and reified a representational landscape that both literally and figuratively erases and dehumanizes Native Peoples. When Native children subsequently look out into their sociocultural contexts, they contend with this legacy and ongoing process of oppression of their Peoples, communities, and ways of being.

Despite settler colonial goals of erasure and dehumanization, being reared in US sociocultural contexts does not have deterministic outcomes for Native children. Child development results from the combination of individual‐level, community‐level, and sociocultural‐level factors (Bronfenbrenner, 1994). As such, while US sociocultural contexts constrain Native children's development, which we will discuss in greater detail below, there exist numerous individual‐, interactional‐, and community‐level protective factors that lead to positive developmental outcomes. For example, at the individual‐level, Native children exhibit immense resilience by constructing personal narratives that connect their past, present, and future together, maintaining optimism about the futures, and advocating for a world in which their identities and experiences are affirmed. At the interaction level, Tsethlikai et al. (2024) provide compelling evidence that parental connection to cultural practices helps to buffer children against the negative physiological and psychological effects of stressful life events. They posit that this is because culturally connected parents actively help children make sense of their experiences in meaningful ways and use more positive disciplinary practices. At the community level, by working to reconstruct familial units after the boarding school era and revitalizing and maintaining Native languages and cultural practices, Native communities help children make sense of their lives and provide the foundations for pride in one's self and group. Ultimately, these individual‐, interactional‐ and community‐level factors make possible a world in which Native children have survived and adapted despite the challenges posed by settler colonialism.

Although Native children and community members have continually navigated and resisted the process of settler colonialism in the service of promoting the positive development of Native children, in the following sections we will highlight the broader sociocultural contexts that work to undermine Native futures. Specifically, we will further detail how contemporary Native children contend with settler colonialism, as it pertains to erasure and dehumanization, in everyday life. We theorize that erasure and dehumanization manifest as acts of omission and misrepresentation in their everyday lives. Next, we discuss the impact of these omissions and misrepresentations on childrens' self and identity development and intergroup relations. Finally, we close with an acknowledgment of the importance of understanding development in context, as the papers in this special issue have done, and the power that the representational landscape can have for promoting paths toward positive child development, greater equity, and ultimately pushing back against the process of settler colonialism.

## OMISSION AND MISREPRESENTATION AS A TOOL OF SETTLER COLONIALISM

When Native children learn about “American” history or look out into contemporary society to see how their group is represented outside of their own communities, they are constantly reminded of the ways in which the United States originated as a settler colony designed to exclude and erase them. They learn about violent and non‐violent efforts to replace, rather than blend with Native nations, to usurp Indigenous lands, and to suppress and destroy Indigenous institutions, practices, and values. The overarching narratives present the actions of settlers and their descendants as necessary for the good of settlers and ultimately for the betterment of the developing nation.

Perhaps most problematic for Native children learning about these historic and contemporary efforts is that they are designed to maintain the settler‐colonist hierarchy (Veracini, 2011; Wolfe, 1999). Specifically, settler colonialism is an ongoing process that shapes relations between colonizers and colonized peoples for generations (Wolfe, 1999). For example, in the late 1800s, under the slogan “Kill the Indian, save the man,” the US government attempted to “Americanize” Native children by placing them in government‐run boarding schools where they were barred from speaking Native languages, practicing cultural traditions, interacting with family and community, and experienced a variety of physical and psychological abuses (Churchill, 2004). Fast forward to the late 19th century, the US government continued its campaign of cultural genocide by forcibly removing Native children from their homes, often with no grounds for removal, and placing them in the care of non‐Native families through foster care and adoption proceedings (Jacobs, 2009). Even though the boarding school era has passed, scientific literature continues to reify the legacy by overlooking and failing to accurately conceptualize the unique ways in which Native Peoples construe caregiving beyond the parent–child dyad, a fact highlighted by Waters et al. (2024). This physical and cultural violence is a continuation of the settler colonial mentality intended to erase Native Peoples and cultures by radically shaping the way Native children were raised. While we are focused on the United States, these processes and dynamics are shared across many other nation‐states such as Canada, New Zealand, Mexico, and Australia. For example, highlighting the consequences of cultural genocide in populations outside of the United States, First Nations children who do not have the opportunity to engage in cultural practices and teachings have worse socioemotional wellbeing and behavioral outcomes compared to First Nations children who do have the opportunity to engage (Owais et al., 2024).

While physical and cultural genocide work to literally erase Native Peoples, another manifestation of settler colonialism works to figuratively erase Native Peoples from public consciousness: what we refer to as *Native omissions*. One of the most commonly discussed Native omissions is the lack of accurate historical accounts of Native people taught in the US schools. Shear et al. (2015) found that 87% of references to Native Americans in the 50 states' academic standards portray Native Peoples in a pre‐1900s context. This means that by and large American children—including Native children—are taught that Native Peoples are people of the past and that contemporary Native Peoples (i.e., “real Natives”) no longer exist. In the rare cases when Native Peoples enter contemporary conversations, they are often portrayed as ‘less‐than’ Native or no longer authentic or “real” Natives.

Furthering the suppression and subjugation of Native children and their tribal nations, mainstream K‐12 curricula promote white‐washed and sanitized versions of US history that fail to acknowledge historical wrongdoings, including longstanding and systematic efforts to eradicate Native Peoples (i.e., genocide), inaccurately portraying relations between Native Peoples and colonists as congenial (e.g., the myth of Thanksgiving or Columbus), and denigrating and undermining complex and knowledgeable Native Nations as “primitive” (Shear et al., 2015). Consider, for example, how the events that unfolded on December 29, 1890, on the Pine Ridge Reservation have been described in classrooms across the United States. The US Army's 7th Cavalry occupied Pine Ridge in an effort to suppress a growing religious movement. The cavalry confiscated the Lakota people's guns and then proceeded to kill between 150 and 300 Lakota, depending on whose estimates one deems reputable, many of whom were women and children. The soldiers received Medals of Honor upon their return home. History textbooks referred to this event as the “Battle” at Wounded Knee. An investigation by the US Army revealed that what happened at Wounded Knee was nothing short of a massacre (Grua, 2015). By describing this massacre as a “battle,” historical accounts omit the US Army's extreme violence against the Oglala Lakota in order to tell a more palatable version of history in which the Lakota and the Army engaged in a fair fight. The more palatable, sanitized version of this narrative has persisted for over 100 years shaping the views and perspectives of multiple generations of American children–including Native children. In 2019, US Representative Deb Haaland worked to get the Medals of Honor rescinded and acknowledge the accurate facts of the massacre. Ultimately, the omission of Native historical perspectives and propagation of white‐washed sanitized versions of history create systematic biases in how American children understand their own history and how they relate to Native Peoples moving forward (see Edmonds, 2010). Similarly, these omissions and misrepresentations suggest to Native children that their group no longer exists, that Native Peoples (if they do exist) lack agency and have limited potential.

The problem of Native omission is not simply relegated to the classroom for Native children. When Native children turn on the TV, watch movies, or play video games they must navigate through a representational landscape comprised of almost no Native characters (i.e., Native characters are less than 1% of all characters) (Lindgren et al., 2023; Nielsen, 2020; Tukachinsky et al., 2015). Even in the rare cases when Native Peoples are represented, the representations are seldom contemporary, diverse, and humanizing. Instead, the representational landscape Native children are exposed to is replete with systematic misrepresentations of Native Peoples that relegate them either to “people of the past” (i.e., omitting contemporary relevance of Native Peoples) or to negative (e.g., bloodthirsty savage) or to romanticized (e.g., spiritual and connected to nature) stereotypes (i.e., omitting humanity, uniqueness, and diversity of Native Peoples; Dai et al., 2021; Davis‐Delano et al., 2021; Fryberg & Eason, 2017; Lopez et al., 2022). Native sports mascot imagery in school, for example, draws on historicized caricatures that either romanticize Native Peoples as brave warriors or derogate them as aggressive savages (Dai et al., 2021; Deloria, 1998; King, 2010).

Hence, when Native children look out in the social world they either do not see themselves or they see a version of themselves that is neither contemporary nor deserving of the same protections as other Americans. For example, protests surrounding police killings of Black Americans have sparked policing reforms at the local, state, and national levels (Grisales et al., 2020). Native Americans face similarly high rates of police violence (Edwards et al., 2019), yet they are largely omitted from discussions about the need for police reform. Similarly, Native communities across the United States are facing an epidemic of missing and murdered Native Peoples. Yet, even with the rise of the #MeToo movement, which brought awareness to issues of sexual assault and created a cultural shift in beliefs about how women should be treated, state and federal officials continue to overlook the ongoing crisis in Native communities. In 2016 alone, the US Department of Justice logged only 116 of the 5712 reported cases of missing Native women and girls (Lucchesi & Echo‐Hawk, 2018; Monchalin et al., 2019). While such omissions systematically obscure urgent problems facing Native Peoples, they suggest to Native children that overt acts of physical and cultural genocide may not be “officially” condoned in the United States, but they still exist and are actively embraced in various domains of US society (Bacon, 2020; Skelley, 2020; Yglesias, 2019).

## PSYCHOLOGICAL IMPACTS OF OMISSION AND MISREPRESENTATION

Given the breadth of omissions and misrepresentations within the representational landscape that Native children must wade through, in the following section, we turn our attention to unpacking the very real social and psychological impacts. Decades of research suggests that social representations help children navigate their social contexts—they help them to develop an understanding of who they are, while also providing non‐Natives scripts for how to engage with them. In the next two sections, we will outline the implications of Native omissions and misrepresentations in two domains: self and identity development and intergroup relations.

### Self and identity development

Starting in infancy, the developing child has a monumental task in front of them: to figure out how to engage with the surrounding world and where they might fit within it. In other words, the questions before children are “who am I,” “where do I belong,” and “who might I become.” The representational landscape is an extremely powerful tool for identifying the answers to these questions, providing potential role models and information about how individuals like them behave toward and are treated by others.

Native omissions and misrepresentations present a challenge for Native children attempting to navigate through the world and develop their sense of self and identity within the broader social and cultural context. Native omissions and misrepresentations limit and negatively impact what Native children see as possible for themselves and what others see as possible for them (Brady et al., 2018; Covarrubias & Fryberg, 2015; Fryberg & Townsend, 2008). Specifically, Native omissions and misrepresentations tacitly (and sometimes not so tacitly) inform Native children that their group is not given the same space, consideration, and concern in various domains that other groups receive (Fryberg & Eason, 2017; Fryberg & Townsend, 2008). For example, in a study of over 6000 Native adults, representing more than 400 tribal nations and all 50 states, 87% of respondents reported feeling that the average American does not care about the experiences of Native Peoples, 77% reported feeling that the average American thinks there are no “real” Native Peoples left, and 75% report that Native people experience discrimination (Findling et al., 2019; Fryberg et al., 2020). A similar pattern emerges among Native children. An independent analysis of data from the 2010 Minnesota Student Survey featuring more than 6000 Native children revealed that only a third of Native children reported that school personnel cared about them “very much” or “quite a bit,” a proportion significantly lower than any other racial/ethnic group (Johnston‐Goodstar & Roholt, 2020). Moreover, in one study, over 98% of college‐going Native students report experiencing at least one microaggression daily (Jones & Galliher, 2015), and in another, 77% of Native children reported being “called a racial slur at school” (Johnston‐Goodstar & Roholt, 2020).

From a social identity perspective, omission, misrepresentations, and ultimately perceptions and experiences of discrimination put Native children in a precarious position as they try to develop their sense of self and identity. How do Native children maintain positive esteem in the face of a social world that is telling them that their group is not worth consideration (i.e., is omitted), has negative traits or “deserves” their poor social outcomes (i.e., negative misrepresentations), and does not exist in contemporary society? Ultimately, how can Native children relate to these representations? Social identity theorizing posits that there are at least two paths that Native children can take in this case: (1) disidentify from the social group (i.e., view being Native as a less important aspect of their sense of self; Becker & Tausch, 2014; Branscombe et al., 2012; Hodson & Esses, 2002; Marshburn & Knowles, 2018; Matschke & Fehr, 2017) or (2) remain identified with their group but attempt to disengage from the social domain that is providing the identity threatening messaging (e.g., disidentify with school; Major et al., 1998; Major & Schmader, 1998; Nussbaum & Steele, 2007).

Disidentification works to put distance between the self and the threatened identity, which can be self‐protective. Nonetheless, ample research demonstrates that social group belonging and identification, broadly construed, has many positive outcomes, including providing people with a sense of meaning (Baumeister & Leary, 1995), while also helping to buffer against the negative impacts of discrimination experiences (Romero & Roberts, 2003; Sellers et al., 2003; Steele et al., 2002; Walters, 1999). Given how widespread omission, misrepresentation, and discrimination are for Native children, such disidentification may leave them vulnerable as they develop a sense of who they are. They may be cutting themselves off from psychologically protective factors against discrimination, including cultural engagement and identification.

For those who remain identified, the journey for self and identity development may be just as challenging. For example, Native children, in the face of narratives of omission and misrepresentation in school report feeling “disengaged” and “alienated” (Journell, 2009, p. 18). School, however, is one of numerous social domains that Native children must interact with on a daily basis. Spaces outside of home and community that affirm their Native identity and provide a sense of belonging are few and far between. The reality is that the representational landscape makes the already difficult task of self and identity development much more treacherous for Native children, resulting in negative psychological outcomes. Specifically, the sense that one's group is omitted and faces discrimination, is related to greater anxiety, depression, negative affect, suicide ideation, and lower life satisfaction (Lopez, Muñoz‐Salgado, et al., 2024; Ward‐Griffin et al., 2024). While the direct evidence on the psychological impacts of omission is more nascent than the data on the impacts of misrepresentations, the picture becomes even more bleak when considering the impact of misrepresentations. The sense that Natives are misrepresented similarly fuels the perception that Natives face discrimination and has negative impacts on anxiety and depression symptomology, negative affect, suicide ideation, and life satisfaction (Lopez, Muñoz‐Salgado, et al., 2024; Ward‐Griffin et al., 2024). Deeper analyses on the effects of exposure to Native mascots, one of the most widespread misrepresentations of Native Peoples, consistently demonstrate that exposure to Native mascots increases experiences of stress, depression, anxiety, and anger, regardless of whether the Native mascot imagery is controversial or not (LaRocque et al., 2011) and decreases the sense of self‐esteem, community worth, and academic aspirations (Fryberg et al., 2008).

Together this data suggest that for Native children the representational landscape, whether it be the media or the educational context, as it stands, is an unwelcome and oftentimes hostile environment that fails to affirm one of the prominent goals of child development: understanding who one is and how to be a self. Instead of affirming children's identity and helping navigate this process as an individual and a social group member, dominant US cultural contexts work to affirm the settler colonial hierarchy which asserts that Native Peoples–past, present, and future–are not worth space, consideration, and concern (Clark & Witko, in press; Fryberg, 2003, 2004; Fryberg & Markus, 2003; Munson, 2001; Society of Indian Psychologists, 1999; Staurowsky, 1999).

### Intergroup relations

While the direct negative effects of the representational landscape on Native children's self and identity development are clear, Native children must also contend with the impacts that the representational landscape has on non‐Native peoples they encounter and engage with. Specifically, social representations theorizing makes clear that the available, or not so available, representations in a given social and cultural context shape the way that people in the context think, feel, and behave (Moscovici, 1984; 1988). Thus, non‐Native peoples are making meaning from the systematic omissions and misrepresentations of Native Peoples (Fryberg et al., 2008; Oyserman et al., 2003).

As a consequence of Native omission and misrepresentation, non‐Natives hold inaccurate and problematic beliefs about Native Peoples (Fryberg & Eason, 2017). For example, more than 40% of non‐Natives explicitly endorse the idea that Native People are “people of the past” (Reclaiming Native Truth, 2018) and over 70% of non‐Natives implicitly hold the same belief (Dai et al., under review). Furthermore, only 34% of non‐Native people believe that discrimination against Native Peoples is still a problem, despite the fact that 75% of Natives report experiencing discrimination (Findling et al., 2019; Reclaiming Native Truth, 2018). Finally, exposure to Native mascots increases the extent to which non‐Natives negatively stereotype Native Peoples as savage, uncivilized, and inferior (Burkley et al., 2017).

By and large, when Native children interact with non‐Native peers and adults, they engage with people who most likely hold limiting and negative beliefs about Native Peoples and communities and are potentially apathetic to their experiences (Dai et al., under review; Davis‐Delano et al., 2020; Lopez, Yellowtail, et al., 2024). In an educational context, these beliefs have particularly damaging consequences for life trajectories. For example, school curricula fail to include–and sometimes actively exclude–Native Americans' cultural history and practices from the learning environment, as these histories and practices are deemed irrelevant to the goals of mainstream education (Quijada Cerecer, 2013). Native students are often perceived to struggle or to be “problem” students (Fryberg & Leavitt, 2014; Johnston‐Goodstar & Roholt, 2020), and they are disciplined more often and more harshly than their White peers (e.g., suspended at more than two times the rate of White peers), despite behaving in comparable ways (Gregory et al., 2010). Conversely, compared with White students with equivalent test scores and grades, teachers are less likely to recommend Native students for advanced coursework (Rowe, 2017). These inaccurate and biased understandings of what is possible for Native students systematically deprive them of the ability to engage with and succeed within a system intended to foster opportunities for upward mobility.

The problem is unfortunately broader than just having to navigate among non‐Native adults who have direct contact with Native children. For non‐Native peoples, the omission and misrepresentation of true history and Native Peoples' experiences helps to maintain a veneer of just and fair treatment (see Fryberg et al., 2024). When individuals do not recognize an oppressive and unjust system as the root of poor outcomes (e.g., disparities in wealth, educational attainment, and incarceration rates), they are likely to justify these outcomes as reflections of the shortcomings of a group, which reinforces negative attitudes and apathy toward that group (Jost et al., 2003; Jost & Hunyady, 2005; Knowles & Lowery, 2012; Young, 1961). Moreover, when individuals see a system as fair and faultless in promoting group‐based poor outcomes, they see no need for systemic solutions (e.g., redistributive social policies, penalties for hate crimes, affirmative actions) to change outcomes (Jost & Hunyady, 2005; Mallett, 2011; Phelan & Rudman, 2011; Sears & Henry, 2005; Wakslak et al., 2007; Yogeeswaran et al., 2018).

The negative impact of omissions and misrepresentations do not just occur as a result of Native children having to engage with non‐Native adults, but negative outcomes also occur in their engagement with peers. Indeed, of the Native children who reported being called a racist slur, approximately equal proportions reported that it came from a peer as those that reported that the slur came from a non‐Native adult. While systematic empirical evidence of non‐Native children's attitudes toward Native Peoples is sparse, emerging evidence suggests that as early as preschool, non‐Native children from diverse racial/ethnic backgrounds feel less positively toward Native children than White children (Keating et al., 2024).

## CONCLUSION: PROMOTING PATHS TOWARD MORE POSITIVE CHILD DEVELOPMENT AND EQUITY

In this paper, we asked how contemporary Native children contend with settler colonialism, as it pertains to the erasure (omission) and dehumanization (misrepresentation) of their social group identities and tribal nations across numerous domains of life (i.e., media and education). We theorize that the ongoing colonization of Native Peoples in the United States continues to influence the self and identity development of Native children around the country. Specifically, we argued that the representational landscape provides unique challenges for Native children as they seek answers to essential developmental questions “who am I,” “where do I belong” and “who might I become.” While the landscape for Native children is treacherous and the data on the impact of erasure and dehumanization is bleak, we want to acknowledge that the representational landscape is in no way deterministic or the fault of Native children. Indeed, as this special issue demonstrates, Native children, Native adults, Native communities, and allied others have worked to expertly navigate through the representational landscape minefield. Efforts to maintain and impart cultural practices and values in early development and throughout the lifespan are just one example of how community and caregivers help children navigate the minefield to promote positive physiological, psychological, and behavioral outcomes (see Owais et al., this issue; Tsethlikai et al., this issue). Native children should not have to live in a world in which, over generations, their unique childhood experiences stemming from traditional cultural practices are increasingly replaced with globalized “best practices” that undervalue Native ways of understanding and engaging in the world. The onus for changing these representations, however, does not rest solely on Native Peoples.

A systemic problem such as the ongoing colonization of Native Peoples requires systemic solutions, which recognize that true inclusion is not just learning *about* Native Peoples but is learning *from* Native Peoples. This starts with refusing to continue the omission and dehumanization of Indigenous Peoples. But beyond this, it requires changing how people think about Native Peoples, creating opportunities and spaces that reflect and uplift Native cultures, understandings, and ways of engagement. To make this change, however, we must first address and change the ways in which education has been used as a tool for colonizing (i.e., erasing and dehumanizing) and/or assimilating Native Peoples. For generations, within Tribal Nations and urban Native communities alike, getting an education has been a metaphor for assimilation or “becoming White” (Davis & Ogbu, 2003; Deloria, 1999; Deyhle & Swisher, 1997; Elliott‐Groves & Fryberg, 2018). Moreover, in the face of increasing globalization, even when this process of assimilation is not explicit, Indigenous child‐rearing practices and their positive cognitive consequences are on the decline across generations (see Rogoff et al., 2024). In seeking to leverage the power of education to address this injustice we, developmental scientists included, must ask some critical questions about the existing educational system: How do the current representations (or lack thereof) of Native Peoples perpetuate the ongoing cycle of dehumanization, and how can we disrupt this cycle? Furthermore, what does it look like to create a socially just and equal educational context for Indigenous students in the United States?

To begin this conversation, we must first contend with what it means to create a socially just and equal educational context. Since the inception of the public education system in the United States, curricula have focused on and bolstered national narratives of progress, expansion, exceptionalism, and modernization that have continuously painted Native peoples as incompatible with these values and thus doomed to vanish entirely (for brief discussion see: Johnston‐Goodstar & Roholt, 2020). Embedded in one of our most long‐standing and widespread American narratives, for example, is the idea that we are a nation that supports “liberty and justice *for all*.” Yet when we look out into our sociocultural contexts, we see stark examples of where we fall short as some groups—such as Native Peoples—are largely absent or omitted from the “for all,” especially when it comes to the historical narratives and social realities shared in school. These omissions and exclusions originate in the nation's founding documents and continue throughout US history, as official national narratives were created to justify physical removal and extermination. So long as Native Peoples lack control of their own representations, they have and will continue to be written out or erased, thereby legitimizing the dispossession of their lands. Creating socially just and equal educational contexts means changing the trajectory of outcomes for not only the current generation of Native children, but for future generations as well. This starts with changing how education is presented to Native children, and ultimately with how Native students experience educational contexts.

The education system is not the only institution that needs to change in order to create and uphold just systems that can support positive developmental trajectories for Native children. The media, for example, can help to intentionally foster new representations and narratives made by Native Peoples that reflect and amplify Native Peoples's voices, experiences, perspectives and values. For children, broadly speaking, the media landscape is already beginning to shift with shows such as Molly of Denali and Spirit Warriors for preschool‐aged children. Similar efforts need to be made by mainstream media to target and develop content for late childhood and early adolescence (approximately 7–15 years of age), and to support developing storytelling among Native children and youth. Finally, although there remains much ground to cover in order to achieve widespread systemic change, the intentional efforts of individuals can have a substantial impact on improving equity for Native Peoples. Indeed, the progress that has been made thus far has undoubtedly come about in part by people choosing to stand with Native Peoples in the work of amplifying Native voices and stories.

Moreover, as developmental scientists, we must take seriously incorporating understandings of the settler colonial goals of erasure and dehumanization when theorizing about child development. Highlighting these broader factors resists deficit frameworks that place the onus on individuals and essentialize negative outcomes. As we consider what it means to promote positive development among Native children, “we can choose to walk forward” as each of the papers in this special issue demonstrates. In doing so, we dedicate ourselves to creating a culture that continues to uplift Native Peoples and communities, to highlighting the benefits of Native practices, and to actively resist Native omissions and misrepresentations to build more equitable and just futures. Alternatively, “we can choose to walk backward” and continue to entrench the process of settler colonialism, which seeks to, yet has arguably failed to, destroy and uproot Native futures. Our hope is that people choose the former path, while recognizing the immense strengths and capabilities of Native children, who have demonstrated resilience and grit in navigating a representational landscape rooted in settler colonialism.
